# Nystagmus in Down Syndrome – a Retrospective Notes Review

**DOI:** 10.22599/bioj.256

**Published:** 2022-06-21

**Authors:** Dilys Oladiwura, yusrah shweikh, Clare Roberts, Maria Theodorou

**Affiliations:** 1 Moorfields Eye Hospital NHS Foundation Trust, GB; 2 National Institute for Health Research Biomedical Research Centre at Moorfields Eye Hospital, GB

**Keywords:** nystagmus, Down syndrome, trisomy 21

## Abstract

**Introduction:**

Nystagmus has been reported in up to 30% of people with Down Syndrome (DS), and yet is still not well understood. Our study aims to characterise the clinical features of patients with DS and nystagmus.

**Methods:**

A retrospective medical-records review was conducted of all patients with a diagnosis of DS and nystagmus seen at Moorfields Eye Hospital over a ten-year period.

**Results:**

Fifty-one subjects were identified, with complete data in 48. The mean age at presentation was 5.1 years (range 0–26 years). The mean binocular LogMAR visual acuity was 0.55(95%CI 0.53–0.57), mean refractive error was –1.8 Dioptre Sphere, DS (95% CI – 5.251.63) with –1.2 Dioptre Cylinder, DC (95% CI – 1.6–0.7). Ocular misalignment was found in 50% of patients. A diagnosis of Fusion Maldevelopment Nystagmus Syndrome (FMNS) was made in 6.3%, Infantile Nystagmus Syndrome (INS) in 8.4% and ABducting nystagmus/Inter-Nuclear Ophthalmoplegia (INO) in 2.1%. The descriptive term ‘Manifest Horizontal Nystagmus’(MNH) was used in the majority, highlighting the difficulties in clinically differentiating the subtypes of nystagmus in DS. Eleven patients had associated cataract. Additional diagnoses unrelated to DS were made in 10.4%.

**Conclusions:**

The most frequent type of nystagmus in our cohort was ‘presumed’ INS. This study highlights the importance of differentiating between FMNS and INS (with a latent component), so that further investigations can be performed as appropriate. Almost 25% had associated cataract, and a further 10% other diagnoses un-associated to DS. Despite INS being known to be associated with DS, further investigations may be required in a small subset with true INS after careful clinical assessment and use of eye movement recordings (where available).

## Introduction

Down Syndrome (DS) originates, in most cases, from a full trisomy of chromosome 21. There has been a steep rise in DS pregnancies due to the rising maternal/paternal age, although the number of live births has fallen (Morris & Alberman 2009). Maternal age is a well-established association but it has also been shown that there is paternal age effect seen in association with a maternal age of 35 years and older, being more pronounced with maternal age of 40 years and older (Fisch et al. 2003). Ophthalmic manifestations are common in DS, including strabismus, refractive errors, and congenital cataract (Akinci et al. 2009). Congenital cataract is also known to be associated with both INS and FMNS (Abadi, Foster & Lloyd 2006; Birch et al. 2012). Subnormal vision is common in trisomy 21 (Zahidi, Vinuela-Navarro & Woodhouse 2018), although this does not necessarily correlate with the ocular findings such as nystagmus (Morton 2011; Felius, Beauchamp & Stager 2014). More recently it has been suggested to be due to retinal developmental changes in DS (O’Brien et al. 2015; Ugurlu & Altinkurt 2020).

In the literature published to date, nystagmus has been described in up to 30% of subjects with DS (Wagner, Caputo & Reynolds 1990; Da Cunha & Moreira 1996). Despite being a well-known association, there will always be practical difficulties in clinically assessing nystagmus in DS, particularly in infants and younger children. Our nystagmus service sees a relatively large number of children and adults with DS and nystagmus, occasionally with alternative/additional diagnoses. These additional diagnoses led to the interest in exploring this area further – the aim of this retrospective notes review was to evaluate our cohort of patients with DS and nystagmus and current ‘standard practice’ as a step towards developing a clinical pathway.

Childhood nystagmus can be broadly divided into two main subgroups: Infantile Nystagmus Syndrome (INS) and Fusion Maldevelopment Nystagmus Syndrome (FMNS). Other subgroups are far less common (Sarvananthan et al. 2009). This terminology was developed by the CEMAS Working Group (2001), whose aim was to provide a foundation of systemic classification of eye movement abnormalities and strabismus that can be used in clinical research. However, despite this classification being published over twenty years ago, in clinical practice, the terms *congenital* and/or *motor nystagmus* are still commonly used interchangeably with INS and similarly with the terms FMNS and (manifest) latent nystagmus. Throughout this paper, we have used the more descriptive terms INS and FMNS.

INS may occur in isolation (‘idiopathic’) where no underlying aetiology can be identified or may be associated with visual afferent pathway abnormalities such as retinal or optic nerve maldevelopment, or any early loss of vision, such as early onset cataract (Bertsch et al. 2017). Early loss of binocular fusion often results in FMNS and is commonly associated with strabismus. There is often a degree of overlap between INS and FMNS – children/adults with INS may have an additional latent component due to poor binocular function, such as co-existing strabismus, and many children/adults with FMNS have a mild manifest component with both eyes open. This overlap makes it more difficult to differentiate clinically with certainty, especially in the distressed child/adult where the external factors can amplify the manifest nystagmus. However, a detailed history adds additional information in addition to the examination. For example, earlier onset nystagmus with definite clinical evidence of binocular function would be more suggestive of INS, whereas a later onset *and* absence of binocular function would be more suggestive of FMNS. In sole FMNS the nystagmus is conjugate, horizontal, and beats in the direction of the viewing eye on monocular occlusion. The nystagmus dampens in adduction (sometimes almost completely) and increases on abduction. INS is typically horizontal, even in upgaze and downgaze. There may be a null region where the nystagmus dampens. A latent component may be seen with an increase in intensity on monocular occlusion. (Self et al. 2020).

The use of eye movement recordings (EMRs) in nystagmus waveform characterisation is well established in both the research and clinical setting. INS is reasonably well understood – There are 12 known waveforms of INS, and the presence of at least one with accelerating exponential slow phase is diagnostic. FMNS slow phases are decelerating exponential (CEMAS Working Group 2001; Dell’Osso & Daroff 1975). However, even with the use of EMRs and interpretation, there remains debate as to the nystagmus subtype in DS (Averbuch-Heller et al. 1999; Felius, Beauchamp & Stager, 2014; Weiss, Kelly & Phillips 2016). In practice, EMRs in a younger, less cooperative child with DS are likely to be noisier and as a result the ‘clean’ data which allows easy differentiation between INS and FMNS based on the characteristics of the slow phase is not often possible. Consequently, increasing awareness of the clinical associations is beneficial to allow safe categorisation rather than simply attributing nystagmus solely to DS.

To date there have been only three studies that have assessed nystagmus in DS with EMRs (nystagmography). There has been disagreement as to whether the nystagmus is FMNS (Averbuch-Heller et al. 1999), INS (Felius, Beauchamp & Stager 2014), or a ‘gaze holding instability’ – a term used by the authors to describe the heterogeneity of the nystagmus and oculomotor changes (Weiss, Kelly & Phillips 2016). Cerebellar hypoplasia is known to be a common finding in Magnetic Resonance Imaging (MRI) of the brain in DS subjects, and may play a role in some (Pinter et al. 2001).

The limited published data suggests that nystagmus in DS is a multifarious entity, with a poorly understood aetiological basis. In the clinical setting children and adults with DS and nystagmus can present to any ophthalmologist. The diagnosis is made on the basis of ophthalmic assessment, and often sub-grouped into one of the two main subtypes. Regardless of which subtype it is presumed to be, if there is no obvious media opacity, the nystagmus is often thought to be directly associated with the DS and not necessarily investigated further. This has implications for the patient and the eventual management if another associated diagnosis is delayed or even missed.

We propose to describe the clinical characteristics of the types of nystagmus associated with DS based on a retrospective notes review in our service and suggest a practical clinical pathway to guide the investigation of nystagmus in patients with DS in the clinical setting.

## Methods

We carried out a retrospective case notes review of all subjects with a diagnosis of nystagmus and DS seen in either the paediatric ophthalmology or adult strabismus services at Moorfields Eye Hospital (London, UK) and its satellite units. The study was approved by the local Clinical Audit Assessment Committee (CA15/ONSP/04).

A search of the electronic records was performed using the terms: ‘trisomy 21’, ‘Down(’s) syndrome’ and ‘nystagmus’ for all patients seen over a 10.5-year period (January 2005–June 2015). Medical records for all the identified patients were reviewed by two clinicians (authors DO and YS).

Clinical details were extracted from the medical records using a standard proforma written by author MT. This included: visual acuity (binocular and, where documented, monocular), manifest/latent strabismus, clinical subtype of nystagmus (by observation only), slit lamp examination of the anterior segment, and dilated fundoscopy. In a subset of patients, further data was collected from supplementary investigations requested by the examining ophthalmologist, such as Electro-Diagnostic Testing (EDT), MRI of the brain and/or Spectral Domain-Optical Coherence Tomography (SD-OCT).

## Results

In total, 51 subjects with DS and nystagmus were identified over the 10.5-year study period. Forty-eight patients were included in the descriptive analysis, and three patients were excluded due to incomplete medical documentation (2 were lost to follow-up and 1 had died). The baseline characteristics and clinical nystagmus subtypes are summarised in Table 1.

**Table 1:** Baseline characteristics of all study participants.


*SUBJECT*	AGE FIRST NOTED (MO)	NYSTAGMUS SUBTYPE	VISUAL ACUITY (BCVA BEO)	REFRACTIVE ERROR (SE)	ASTIGMATISM (>0.75D)	STRABISMUS	CATARACT	OTHER FINDINGS OF NOTE

1	<1	INS	HM	High myopia	Yes	LET	Yes	Bilateral lensectomy/aphakia, Secondary glaucoma

2	<1	INS	0.5	High myopia		No	Yes	Blue dot cataracts

3	18	MHN (FMNS)	0.50	Moderate hyperopia	Yes	AET		Bilateral ptosis

4	2	MHN (INS)	0.700	High myopia	Yes	AET		Bilateral optic nerve and retinal coloboma. Normal MRI

5	<1	MHN (INS)	0.800	Moderate myopia	Yes	No	Yes	Blue dot cataracts

6	<1	MHN (INS)	0.5	High myopia	Yes	No	Yes	Mild cataracts

7	<1	MHN (INS)	0.500	High myopia	Yes	No		Keratoconus

8	<1	MHN (INS)	0.2	Moderate hyperopia	Yes	No	Yes	Mild cataracts

9	6	MHN (INS)	0.4	Moderate myopia	Yes	No		

10	<1	INS	0.64	High myopia	Yes	AET		

11	<1	MHN (INS)	F+F	High myopia		LET	Yes	Bilateral lensectomy/lens implant, Left funnel retinal detachment

12	<1	MHN (INS)	0.35	Low hyperopia	Yes	No		

13	<1	MHN (INS)	1.3	Moderate myopia	Yes	RXT	Yes	

14	U	MHN (INS)	0.5	-	-	No		

15	<1	MHN (INS)	0.725	Moderate myopia	Yes	No		

16	3	MHN (INS)	0.24	Low hyperopia	Yes	AET		

17	36	MHN (INS)	1.00	High myopia	Yes	No		

18	4	MHN (INS)	0.6	Moderate hyperopia	Yes	AET		

19	<1	MHN (INS)	0.475	Low myopia	Yes	No		

20	<1	MHN (INS)	0.6	Low hyperopia	Yes	No		

21	Unknown	MHN	0.275	Low myopia	Yes	AXT		

22	<1	MHN (INS)	1.0	Moderate myopia		No	Yes	Achromatopsia (CNGB3 mutation)

23	2	MHN (INS)	0.320	Moderate myopia	Yes	RET		

24	<1	MHN (INS)	0.9	Low hyperopia	Yes	RXT		Achromatopsia

25	6	MHN (FMNS)	0.5	Low hyperopia		ET		

26	4	INS	0.4	Low hyperopia	Yes	No		

27	<1	MHN (INS)	0.1	Moderate hyperopia		No		

28	4	MHN (INS)	0.4	Moderate myopia	Yes	No		Unilateral optic nerve hypoplasia. Normal MRI

29	6	MHN	0.5	Low myopia	Yes	AET		

30	Unknown	FMN	0.275	No significant error	Yes	RET		

31	<1	MHN (INS)	0.2	No significant error		RET		

32	Unknown	FMN	0.30	Moderate hyperopia	Yes	No		

33	<1	MHN (INS)	0.3	Hyperopia	Yes	No		

34	3	MHN (INS)	0.6	Low hyperopia	Yes	ET		Post retinal dysfunction (EDTs). MRI-hypoplastic inferior cerebellar vermis and pons

35	4	MHN (INS)	F+F	Moderate hyperopia		No		VEPs – Chiasmal misrouting

36	<1	MHN (INS)	0.5	No significant error		RXT		

37	2	MHN (INS)	0.500	Hyperopia		No		

38	6	MHN	0.5	High hyperopia		LET	Yes	Bilateral lensectomy/aphakia

39	6	MHN	0.8	Hyperopia Yes		ET		

40	12	MHN	0.60	High myopia	Yes	RET		

41	14	MHN (INS)	F+F	Low hyperopia	Yes	No		

42	3	MHN (INS)	0.800	Myopia		LET	Yes	Bilateral lensectomy/aphakia

43	2	MHN (INS)	1.00	Severe hyperopia	Yes	LET	Yes	Bilateral lensectomy/aphakia, Bilateral keratoconus

44	4	MHN (INS)	1.00	High myopia	Yes	No		

45	6	MHN (INS)	0.5	Hyperopia	Yes	No		

46	3	MHN (INS)	0.6	Moderate hyperopia	Yes	No		

47	6	FMN	0.6	Moderate hyperopia	Yes	No		

48	Adult	INO	0.2	Moderate hyperopia	Yes	LXT		


Key: mo-months; INS – Infantile Nystagmus Syndrome; MHN – Manifest Horizontal Nystagmus; FMNS-Fusion Maldevelopment Nystagmus Syndrome; BCVA BEO – Best Correct Visual Acuity Both Eyes Open; F+F – Fixing and Following; HM – Hand Movements; SE-Spherical Equivalent; L/R/A – Left/Right/Alternating (L/R/A); ET – Esotropia; XT – Exotropia; EDTs – Electro-Diagnostic Tests; MRI -Magnetic Resonance Imaging.

The mean age of presentation to our service was 5.1 years (range 0–26 years) with 56.3% of the patients being female. The average reported age at which nystagmus was first noticed was 6.1 months (95% CI 2.2–10.0 months).

The mean LogMAR visual acuity with both eyes open was 0.55 (95% CI 0.53–0.57). A variety of visual acuity tests were used depending on the chronological/developmental age. The mean refractive error was –1.81 DS (95% CI –5.25 – –1.63D) with –1.17 DC (95% CI –1.63 – –0.71D). Primary ocular misalignment was documented in 50% (24/48) of subjects: 75% (18/24) of strabismic patients had an esotropia and 25% (6/24) an exotropia. Vertical misalignment in the primary position was not documented in this cohort.

In the majority of notes, a clinical diagnosis of ‘Horizontal Manifest Nystagmus’ was documented in 40/48 (83.3%) – the use of this descriptive term was considered to be due to the inability to differentiate further clinically due to the difficulty with the assessment. We categorised the diagnosis as ‘presumed INS’ in 33/40 (82.5%) if there was: a documented early onset (<6 months) or if there was definite clinical evidence of binocular function, and as ‘presumed FMNS’ in 2/40 (5%) based on a later onset (≥6 months) AND absence of binocular function, such as presence of manifest strabismus*. Where no further classification was possible based on the notes review, this was documented (5/40,7.5%). A clinical notes diagnosis of INS had been made in 4/48(8.3%), and a primary diagnosis of FMNS in 3/48 cases (6.3%).

*This is not a validated classification, but due to the limitations of a retrospective study, a common sense clinical approach to reviewing the data was taken

In 1/48 (2.1%) the nystagmus was clinically neither INS nor FMNS: A 26-year-old male patient with a Parkinsonian syndrome presented with an ABducting nystagmus (as part of an Inter-Nuclear Ophthalmoplegia (INO)). This patient was found to have bilateral basal ganglia iron deposition on an MRI of the brain. Of note, 11/48 patients had documented cataract, with 6/11 requiring surgical intervention in infancy/childhood.

EDTs were requested in only 18/48 (37.5%) of patients, although the reasons for requesting these was not documented in the clinical notes. Definitive abnormalities in EDTs were found in 5/18 cases. These could be grouped into: optic nerve dysfunction, which was found in 2/18 (11.1% of those formally tested, 4.2% of group); cone dysfunction in 2/18 (11.1% of those formally tested, 4.2% of group); and chiasmal misrouting on visual evoked potentials (VEPs) in 1/18 (5.6% of those formally tested, 2.1% of group).

Of the two children with optic nerve dysfunction, one child had bilateral optic nerve and choroidal colobomata; and one child had unilateral optic nerve hypoplasia, both seen on clinical examination. Neither had any other associated neurological comorbidities found on MRI of the brain, orbit, optic nerves, chiasm, and visual pathways.

The two children found to have poor cone function on Electro-Retinogram (ERG) both presented with additional symptoms of photophobia and markedly reduced documented visual acuity. SD-OCT imaging did show bilateral mild outer retinal disruption of the maculae. This was attributed to achromatopsia in both children. Molecular genetic testing confirmed the CNGB3 gene in one, but did not identify any of the known mutations in the other.

The child with chiasmal misrouting documented on VEPs had clinical signs in keeping with albinism, including iris transillumination, reduced fundal pigmentation and reduced foveal reflexes. EDTs confirmed a contralateral predominance on flash VEP examination, which is a common finding in the majority of patients with albinism. One child with possible ‘post-retinal dysfunction’ underwent neuro-imaging of the visual pathways, with no abnormality documented.

In total, 4/48 patients underwent neuro-imaging (all by MRI) based on clinical and/or EDT findings as already described above.

## Discussion

The main findings of this small retrospective study suggest that nystagmus in children and adults with DS may be more heterogeneous than previously thought, that is, not always simply ‘associated with DS’ as may be assumed by many clinicians. The subtypes of nystagmus in infants/children/adults may be difficult to differentiate on the basis of clinically observed waveform pattern alone, particularly in the paediatric population, and those with significant developmental delay. EMRs, although well established, may be more difficult to obtain in practice, with significant noise/artefact, precluding waveform analysis. It is therefore prudent to take into consideration the full context in which a patient with DS presents, as in other patients with nystagmus, and review each case with an individualised approach.

In a child (with or without DS) an onset before six months of age of bilateral, conjugate predominately or purely horizontal nystagmus, can indicate a broad initial diagnosis of INS on the basis of these features. When patients or families are uncertain of the age of onset, it may be more difficult to differentiate the subtype based on clinical assessments alone, in the absence of definite clinically localising signs such as ABducting nystagmus as part of an INO which would direct the clinician towards a neurological/non-ocular aetiology). EMRs (where tolerated and compliance allows) may help differentiate the benign forms INS, INS with latent component and FMNS from other subtypes which could require more urgent investigation where this is not identified clinically (CEMAS Working Group 2001).

Differentiation between a decelerating and accelerating exponential slow phase waveform to confirm FMNS and INS is well documented and increasingly utilised where access allows (CEMAS Working Group 2001; Dell’Osso & Daroff 1975), so will not be discussed in further detail here. In an ideal world, EMRs and analysis would be readily available in all units, although a certain degree of compliance is required, even for the experienced observer, to allow quantitative and qualitative analysis. However, across many healthcare systems worldwide access to EMRs may be constrained, either by financial resources or by access to expertise required to obtain and interpret EMRs, particularly for those patients whose compliance with recording may not be optimal due to their learning disability.

The study, although subject to the many limitations of retrospective case notes review, serves as a reminder of the everyday difficulties encountered when assessing the child with nystagmus and DS. Many were documented simply as having ‘manifest horizontal nystagmus’, suggestive of the difficulties in clinical assessment. This non-specific term does not allow differentiation of those that need further investigation and management. Initial clinical assessment should involve a step-wise approach, as for any other infant or child presenting with nystagmus, to exclude a co-existing ocular or non-ocular condition. This should start with a detailed history followed by orthoptic assessment and ocular examination. Clinical examination should highlight structural abnormalities such as media opacity, including corneal scars or congenital cataracts, which may be present in up to 2.8% of children with DS (Haargaard & Fledelius 2006) as well as high refractive errors which can also cause visual deprivation. Visual deprivation may also occur in the presence of albinism, retinal disease and optic nerve pathology (Akinsi et al. 2009; Barrett, Bradley & Candy 2013). A small proportion of the cases in our cohort required several levels of investigations to elucidate the presence of other pathology unrelated to DS based on the clinical picture. Of note, other than lens opacity, none of the ocular comorbidities detected in our cohort (5/48, 10.4%) were associated with chromosome 21 – that is, they were completely separate entities.

Accommodative and refractive errors, as well as emmetropisation in DS have been well described (Al-Bagdady, Murphy & Woodhouse 2011; Cregg et al. 2001; Woodhouse et al. 1997; Hashemi et al. 2021) and although it is not the focus of this paper, it was noted that the mean level of astigmatism in this group was lower than previously described. The cause of this is unclear – Asgari et al. (2021) suggested that in non keratoconic DS patients corneal power causes against-the-rule astigmatism, so this may be partly balanced with the with-the-rule astigmatism associated with horizontal nystagmus (Wang et al. 2010), although Little et al. (2009) did not find a relationship between corneal power and astigmatism in DS.

It is well documented that visual acuity and contrast sensitivity (when measured either with behavioural methods and/or visual evoked potential) is considerably lower in children with DS even when ophthalmic abnormalities have been excluded (John et al. 2004; Suttle & Turner 2004). In comparison with published normal for various acuity tests, binocular visual acuity in DS is thought to stabilise at around 0.25 LogMAR from the age of four years (Zahidi, Viinuela-Navarro & Woodhouse 2018). In our study the mean LogMAR Visual Acuity with both eyes open in subjects with DS and nystagmus was further reduced at 0.55LogMAR (95% CI 0.53 to 0.57). Best corrected visual acuity lower than this (following for a period of refractive adaptation), even in the absence of abnormal clinical findings, should raise the index of suspicion of other associated co-morbidities this cohort. With the exception of the subject with unilateral optic nerve hypoplasia, our cohort with abnormal findings on EDTs all had severely reduced visual acuity (worse than 0.7LogMAR). Electrodiagnostic testing in our study proved to be an important tool for identifying specific coexistent diagnoses in this subset of patients with DS who present with nystagmus with poor visual acuities. During the period covered by the study, OCT has become both higher quality and more readily available. This may allow earlier identification with of diagnoses such as albinism and achromatopsia where foveal changes have been well documented (Lee et al. 2013; Sundaram et al. 2014).

Although nystagmus in DS is commonly termed ‘idiopathic’, that is, having no other associated ocular/neurological co-morbidities, our cohort has shown that other coexisting pathologies both related and unrelated to DS can be present in both children and adults with early onset nystagmus, without any documented association to DS. Since we serve as a tertiary referral centre for many paediatric subspecialties we may have a skewed representation, for example, congenital cataracts may be disproportionately represented in our case series. Nevertheless, it is important to be aware of associations in nystagmus and DS, as identifying these conditions is a pivotal step for devising a holistic approach for the management of children with DS. Children with DS often have other difficulties which may be compounded by unrecognised visual problems, which have implications for learning, cognitive functioning, and adaptive behaviour. Visual impairment in individuals with intellectual disability is associated with higher levels of maladaptive behaviours in comparison to their counterparts without intellectual disability (Prasher 1994). Any associated diagnoses need to be managed appropriately. In particular where an unrelated genetic cause for nystagmus is diagnosed, far reaching implications may result as it pertains to future pregnancies for the family, the learning needs of the child and availability of support, over and above the diagnosis of DS alone. A multidisciplinary approach may be required to ensure that children and adults reach their full potential and parents/guardians receive the support that is required to allow this.

**Figure 1:**
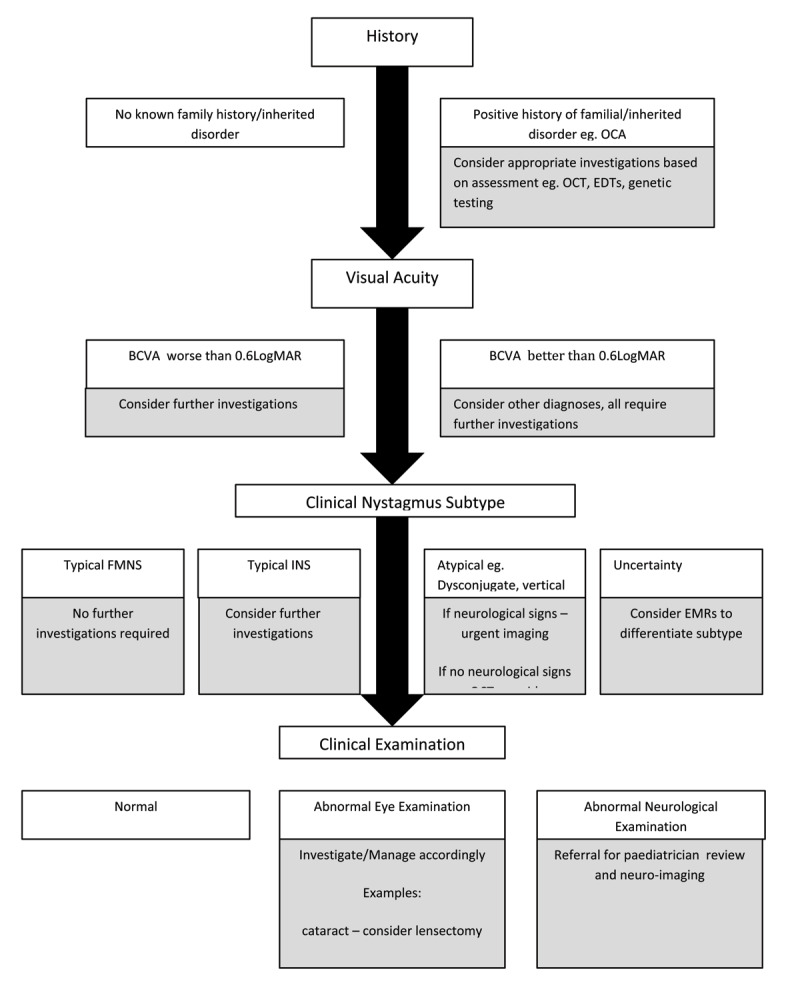
Proposed clinical pathway for investigation of nystagmus in patients with Down’s Syndrome. Key: BCVA – Best Corrected Visual Acuity; EDTs – Electro-Diagnostic Tests; EMRs – Eye Movement Recordings; FMNS – Fusion Maldevelopment Nystagmus Syndrome; INS – Infantile Nystagmus Syndrome; OCA- Oculo-Cutaneous Albinism; OCT – Optical Coherence Tomography.

A step-wise approach in assessing and investigating nystagmus in the context of DS in both specialist and non-specialist units may be useful, as electrodiagnostic testing and eye movement recording may not be readily available. A suggested clinical pathway is illustrated in Figure 1. Although this is a simplified schematic of nystagmus in DS, it provides a guide to initial management, which can be utilised in most clinical settings.

Although this small study is limited by its retrospective nature, it highlights the variability of nystagmus in DS in the typical clinic, and that each child/adult should be managed as an individual and further investigated where appropriate. In keeping with previous literature, the most frequent type of clinically documented nystagmus in our cohort was presumed INS, although it is often difficult to clinically differentiate between FMNS and INS with a latent component. Differentiating between the subtypes, based on clinical assessment with targeted further investigations can inform decisions to be made regarding further investigation and or future management, allowing early support and rehabilitation.
